# A review of feeding methods used in the treatment of anorexia nervosa

**DOI:** 10.1186/2050-2974-1-36

**Published:** 2013-09-02

**Authors:** Susan Hart, Richard C Franklin, Janice Russell, Suzanne Abraham

**Affiliations:** 1 Department of Psychiatry, Royal Prince Alfred Hospital, Camperdown, NSW 2050, Australia; 2 Boden Institute of Obesity, Nutrition, Exercise and Eating Disorders, The University of Sydney, Camperdown, NSW 2006, Australia; 3 School of Public Health, Tropical Medicine and Rehabilitation Sciences, James Cook University, Townsville, QLD 4811, Australia; 4 Northside Clinic Eating Disorder Program, Greenwich, NSW 2065, Australia; 5 Department of Obstetrics and Gynaecology, Royal North Shore Hospital, St Leonards 2065, NSW, Australia

**Keywords:** Anorexia nervosa, Feeding, Enteral, Nasogastric, Refeeding, Parenteral

## Abstract

**Background:**

Clear evidence based guidelines on the best and safest method of achieving and maintaining normal body weight during inpatient treatment of Anorexia Nervosa (AN) are currently not available. Oral feeding with food alone, high-energy liquid supplements, nasogastric feeding and parenteral nutrition all have the potential to achieve weight gain in the treatment of AN but the advantages and disadvantages of each method have not been comprehensively evaluated.

A literature search was undertaken to identify papers describing feeding methods used during inpatient treatment of AN. The selection criteria searched for papers that described the feeding method; and reported weight change variables such as admission and discharge weight in kilograms, or Body Mass Index; or weight change over the course of inpatient treatment.

**Results:**

Twenty-six papers were identified, describing a total of 37 samples with a mean sample size of 58.9 participants, and a range from 6 to 318. The majority (84.6%) of papers were observational cohorts and retrospective chart reviews. The most common feeding method described was nasogastric feeding and food, then high-energy liquid supplements and food.

**Conclusions:**

There is limited evidence on the efficacy of feeding methods used in the refeeding and nutritional rehabilitation of AN, therefore no conclusion can be made about the most effective method of achieving weight gain during inpatient treatment. While there are a number of papers exploring this issue there is no consistency in the way the information is reported to enable comparisons between the different methods. There is an urgent need for research in this area to guide decision-making in the inpatient management, refeeding and nutritional rehabilitation of AN.

## Background

Under-nutrition and its sequelae are a major consequence of Anorexia Nervosa (AN) with refeeding and restoration of nutrition status a primary aim of the initial treatment. Guidelines on the treatment of AN [1-6] highlight the importance of nutrition intervention to restore weight, and help patients normalise their eating. However, clear evidence based guidelines on the best and safest method of achieving normal body weight during inpatient treatment, are currently not available.

There are several feeding methods used as part of the refeeding process in AN, such as with food alone, using high-energy liquid supplements, nasogastric (or enteral) feeding, and parenteral nutrition [7-10]. This review defines “food only” to be when all energy and nutrients are provided by whole food alone. “High-energy liquid supplements” are drinks, which provide energy and micronutrients delivered in portion-controlled packaging. “Nasogastric feeding” is where nutrients are delivered directly into the gastrointestinal tract with a thin plastic tube through the nose, and the tip of the tube sitting in the stomach, duodenum or jejunum [11]; energy and micronutrients are provided as a liquid formula delivered via the tube. “Parenteral nutrition” provides elemental nutrition in solution, in the form of amino acids, lipids and dextrose, which are delivered directly into the systemic circulation by a peripheral or central vein, bypassing the gastrointestinal tract [12-14].

Liquid supplements, nasogastric feeding and parenteral nutrition have the potential to provide one hundred percent of an individual’s recommended daily intake of nutrients or may be used to provide additional energy to any food that is eaten, in order to meet nutritional requirements and assist with weight gain. Nasogastric feeding and parenteral nutrition have an established use in acutely ill hospitalised patients who are unable to eat secondary to their illness or injury [15,16]. All four methods have the potential to achieve weight gain in the treatment of AN but the benefits and adverse effects of each method have not been comprehensively evaluated when used as a treatment strategy for AN.

This paper aims to review the published peer-reviewed literature on the feeding methods used in the inpatient treatment of AN, and to report any advantages and disadvantages of each method. Additionally, the authors aimed to determine if there is evidence of one feeding method being superior to another in terms of weight gain during inpatient treatment.

## Methods

A keyword search of the databases Medline and PsycINFO was undertaken between June and December 2012, to identify papers describing feeding methods used during inpatient treatment of AN. Five searches were undertaken using the key word “anorexia nervosa” combined with a second key word. The second key words used for the literature search, when combined with “anorexia nervosa”, were “nasogastric”, “enteral”, “feeding”, “parenteral” and “refeeding”. Additionally, a hand search was undertaken of key eating disorder journals as was snowballing of the published literature to identify any additional papers. The selection criteria for this review were papers which: described inpatients with a diagnosis of AN; provided a description of the feeding method used; and included weight change variables such as admission and discharge weight in kilograms, or Body Mass Index (BMI), or weight change over the course of inpatient treatment. Articles meeting the criteria (N = 26) were reviewed for study design, sample size, age of participants, length of stay, and prescribed energy intake. Papers that did not meet the inclusion criteria but described benefits or adverse effects of a feeding method were identified and pooled.

Papers were excluded if: they were case studies with less than 10 participants; there was no description of the feeding method used; it was not clear which feeding method was being used; the subjects were not inpatients or were combined cohorts of inpatients and outpatients and unable to be separated; or it did not report weight change, or admission and discharge BMI. Only articles in English were reviewed. The results were tabulated in PASW Statistics GradPack 18.0*.*

## Results

In this literature search of feeding methods and inpatient treatment of AN, 26 papers were identified that met the inclusion criteria (Table 1). As 10 papers also included a comparator group (with one paper having two comparator groups), the search resulted in a total of 37 samples (Table 2).

**Table 1:** Papers meeting the inclusion criteria

**Author**	**Study design**	**Method**	**Sample size**	**Age (years)**	**Admission BMI (kg/m**^**2**^**)**	**Discharge BMI (kg/m**^**2**^**)**	**Change in weight (kg)**	**Length of stay (days)**	**Prescribed energy intake (kCal)**
***N = 26***	***n = 26***	***n = 37***	***n = 37***	***n = 31***	***n = 31***	***n = 24***	***n = 31***	***n = 27***	***n = 16***
Castro-Fornieles, [10]	Observational	Food	49	14.4	15.5	18.4	8.7	29.8	-
Castro, [17]	Observational	Food	101	14.9	15.9	-	6.3	31.2	-
Courterier, [18]	Chart review	NG + food	12	15.7	-	-	5.5	78.0	-
Dalle Grave, [19]	Chart review	Food	35	22.3	12.6	-	10.1	92.4	-
Diamanti, [8]	Chart review	PN + food	104	14.9	14.3	15.6	3.1	30.7	2175
		Food	94	15.2	16.0	16.3	1.2	15.6	2078
Garber, [20]	Observational	SUP + food	35	16.2	16.3	-	2.4	16.7	2668
Gentile, [21]	Observational	NG + food	33	22.8	11.3	13.5	5.4	-	1736
Gentile, [22]	Chart review	NG + food	75	16.8	12.6	18.3	14.9	-	-
Hart, [23]	Observational	SUP + food	96	-	15.1	16.9	4.2	48.7	2520
		Food	318	-	16.6	17.8	3.3	38.0	1980
Imbierowicz, [7]	Chart review	SUP + food	42	24.8	15.2	-	5.2	72.8	-
		Food	42	23.7	15.3	-	3.5	82.6	-
Krahn, [24]	Observational	Food	10	-	15.4	19.4	10.4	-	3600
Lay, [25]	Chart review	Food	40	15.2	13.6	17.2	9.4	141.0	-
Lund, [26]	Prospective	NG + food	79	21.6	16.3	20.8	12.2	103.4	-
Neiderman, [27]	Survey	NG + food	19	13.6	15.4	17.5	-	-	-
Okamoto, [28]	Observational	SUP + food	21	20.5	13.5	16.7	-	108.9	-
		Food 1	7	18.4	12.9	15.7	-	149.0	-
		Food 2	7	18.2	14.5	16.0	-	70.4	-
Ornstein, [29]	Chart review	NG + food	69	15.5	15.0	16.9	4.9	25.6	-
Ostuzzi, [30]	Observational	SUP + food	53	24.7	-	-	6.2	101.9	-
Pertushuk, [31]	Chart review	PN + food	23	25.4	-	-	2.5	62.6	-
		Food	136	23.8	-	-	1.1	29.6	-
Rigaud, [32]	RCT	NG + food	19	-	15.8	17.4	-	-	1832
		Food	17	-	16.2	16.6	-	-	1642
Rigaud, [9]	RCT	NG + food	41	22.5	12.1	17.9	9.6	-	-
		Food	40	24.2	12.8	15.9	5.0	-	-
Robb, [33]	Chart review	NG + food	52	14.8	15.5	17.5	5.4	22.3	3255
		Food	48	15.0	16.0	16.8	2.4	22.1	2508
Silber, [34]	Chart review	NG + food	6	13.8	15.3	19.1	10.9	36.0	4350
		Food	8	14.9	17.4	18.5	3.8	39.9	3400
Stordy, [35]	Observational	Food	11	19.9	-	-	12.8	-	2800
Tonoike, [36]	Chart review	PN + food	46	21.0	12.7	17.2	11.1	63.5	-
Walker, [37]	Observational	SUP + food	29	-	-	-	8.4	-	-
Zeurcher, [38]	Chart review	NG + food	155	25.7	14.2	-	8.1	61.0	3035
		Food	226	25.2	15.7	-	5.7	48.3	2815

Food = Food only; SUP = High-energy liquid supplements; NG = Nasogastric feeding; PN = Parenteral nutrition; RCT = Randomised controlled trial.

**Table 2:** Summary of results

	**Food only**	**SUP + Food**	**NG + Food**	**TPN + Food**	**Total**
N papers total	6	6	11	3	26
Total samples (including 10 comparator samples; n)	17	6	11	3	37
Papers with the primary aim to describe the feeding method?	0	3	8	3	14
Mean sample size (range)	69.9	46.0	50.9	57.7	59.4
	(7 – 318)	(21 – 96)	(6 – 155)	(23 – 104)	(6 – 318)
	n = 17	n = 6	n = 11	n = 3	n = 37
Total number of participants for method	1189	276	560	173	2198
Mean length of stay in days (range)	60.7	69.8	54.4	52.3	60.1
	(16–149)	(17 – 109)	(22 – 103)	(31 – 64)	(16 – 149)
	n = 13	n = 5	n = 6	n = 3	n = 27
Mean admission BMI (kg/m^2^) (range)	15.1	15.0	14.4	13.5	14.7
	(12.6 – 17.4)	(13.5 – 16.3)	(11.3 – 16.3)	(12.7 – 14.3)	(11.3 - 17.4)
	n = 15	n = 4	n = 10	n = 2	n = 31
Mean discharge BMI (kg/m^2^) (range)	17.4	16.8	17.7	16.4	17.4
	(15.7 – 20.3)	(16.7 – 16.9)	(13.5 – 20.8)	(15.6 – 17.2)	(13.5-20.8)
	n = 12	n = 2	n = 9	n = 2	n = 25
Mean total weight gain in kilograms (range)	6.0	4.3	8.4	7.1	6.7
	(1.1 – 12.8)	(2.4- 6.2)	(4.9 – 14.9)	(3.1-11.1)	(1.1 – 14.9)
	n = 11	n = 3	n = 8	n = 2	n = 24
Mean energy intake (kCals) (range)	2603	2594	2842	2175	2650
	(1642–3600)	(2520–2668)	(1736–4350)	-	(1642–4350)
	n = 8	n = 2	n = 5	n = 1	n = 16

Food = Food only; SUP = High-energy liquid supplements; NG = Nasogastric feeding; PN = Parenteral nutrition.

Search one of “nasogastric” and AN identified 23 papers, of which 10 were reviewed, and seven were kept. Four of these seven papers included a comparator group. Reviewed papers were excluded because they were case series with less than 10 participants (n = 2), and did not describe any weight data (n = 1). Search two of “enteral” and AN identified 104 papers; 17 were reviewed and three were kept. Two of these three papers included a comparator group. Reviewed papers (n = 14) were excluded because they were case studies with less than 10 participants (n = 8), had insufficient weight data (n = 3), were a descriptive paper (n = 1), and presented data that could not be extracted (n = 2). Search three of “feeding” and AN identified 613 papers. Thirty-two papers were reviewed and three were kept. Two of the three papers included a comparator group. Reviewed papers were excluded because they had insufficient weight data (n = 10), were case series with less than ten participants (n = 2), did not adequately describe the feeding method (n = 13); or were review studies (n = 4). Search four, which used the keywords “parenteral” and AN identified 101 papers. Thirteen were reviewed, one was kept, which did not have a comparator group. Reviewed papers were excluded because they were case series with less than 10 participants (n = 5), had insufficient detail of the feeding method (n = 1) or were descriptive papers with no data (n = 6). Search five, using the keywords “refeeding” and AN identified 279 papers. Eleven were reviewed, and two were kept. Nine reviewed papers were excluded because there was insufficient detail on weight variables (n = 3), they were case series with less than 10 participants (n = 3), had insufficient detail on the feeding method (n = 1), the feeding method was not clear (n = 1), and described pooled data from 30 separate treatment facilities (n = 1). None of these papers had a comparator group. Ten relevant papers were identified using the hand search and snowballing methods, of which one paper had a comparator sample, and one paper had two comparator samples.

The majority (84.6%) of papers were observational cohorts (n = 10) and retrospective chart reviews (n = 12). Two papers were randomised controlled trials, both by the same author, and one study had a prospective longitudinal design. Numerical data was extracted for age, sample size, admission and discharge BMI, total weight change in kilograms, prescribed energy in kilocalories, and length of stay in days, and are tabulated for each paper (Table 1), and pooled with means and ranges calculated (Table 2). The mean sample size for each of the feeding methods was 58.9 participants, with a range of 6 to 318. The method that was used for the greatest number of participants (n = 1189) was for food only samples. The greatest mean weight change was 8.4 kilograms (kg) for nasogastric feeding and food, then parenteral nutrition and food (7.1 kg), then food only (6.0 kg) and liquid supplements and food (4.3 kg). As the reviewed papers that the data was extracted from were predominantly observational and retrospective chart reviews, no further analysis was undertaken.

Fourteen out of 26 papers meeting the inclusion criteria had the description and evaluation of a feeding method as its main aim. Only 16 of 37 samples reported prescribed energy intake, and only 27 of 37 samples reported length of stay. Only three papers of 26 (11%) reported numerical data for all seven variables (i.e. sample size, age, admission and discharge BMI, weight change, length of stay and energy intake). The most frequent feeding method described was nasogastric feeding and food, then liquid supplements and food. There were only three studies meeting the inclusion criteria that described the use of parenteral nutrition. Food only was described in six studies where it was the only method of refeeding however in 11 papers, food was the comparator group for the other feeding methods, resulting in a total of 17 food only samples. Most information about the benefits and adverse effects of the feeding methods were extracted from descriptive papers, which are pooled in Table 3.

**Table 3:** Benefits and adverse effects of four feeding methods

**Food only feeding**	
**Benefits**	**Adverse effects**
• It teaches skills for eating, promotes normal behaviour, and challenges unhelpful coping strategies [39];	• Less energy is delivered from food when compared with nasogastric feeding [9].
• Patients experience the amount of food necessary for weight gain and weight maintenance [40];	
• Food makes hospital meal management home-like and realistic, which exposes patients to a situation which is anxiety-provoking, and gives them confidence at managing meals at home [41].	
**High-energy liquid supplements**	
**Benefits**	**Adverse effects**
• Supplements can meet the high-energy requirements required for weight gain in a smaller volume than food [7,42];	• The frequent use of supplements encourages patients away from the experience of food, re-enforces their avoidance of food and can foster dependency on artificial food sources [39].
• They are helpful as a “top-up for patients struggling with satiety and the quantities of food required to promote weight gain [39,40];	
• It can be seen as a type of medicine [43].	
**Nasogastric feeding**	
**Benefits**	**Adverse effects**
• More comfortable for the patient with less pain, physical discomfort and abdominal distension than large amounts of food [33,34,38].	• It interferes with the fragile alliance between the patient and treatment team [44];
	• The patient may feel disempowered and embittered towards the treatment team, which may have an impact on future personal and professional relationships [45];
• A helpful strategy aiding recovery:	
o It transfers the responsibility of weight gain from the patient to the treatment team [46];	
		• It is invasive, frightening, unpleasant and mirrors the dynamics of trauma [27,39];
o If placed upon admission, it “medicalises” the treatment, and reduces the “power struggle” between the patient and clinicians [34].		
	• There is an emotional toll on staff treating involuntary patients [18];	
• Opinions from patients and carers:	• Not helpful for long term recovery:	
o Nasogastric feeding was seen as necessary by some patients because they believed they lacked the physical or psychological capacity to eat [47];	o Patients may demonstrate an inability to maintain adequate intake and weight gain once the tube is removed [9,46];	
o Parents recognized it as a last resort that was required to keep their child alive [27];	o Force feeding in low weight patients achieved little in relation to remitting illness or suffering [48];	
o It reduced the pressure patients perceive is being placed on them to eat and temporarily relieves responsibility for adopting improved eating behaviours [47]	o Patients tamper with the tube by adjusting the control, decanting the feed into other containers when unobserved, biting, and removing the tube [27,32,33,40,48].	
	• Medical complications i.e. aspiration [49]; nasal bleeding and nasal irritation [9,18,33]; reflux and sinusitis [9,32];	
	• The tube may not be inserted properly which is more likely when patients have one inserted against their will [40];	
	• Opinions from patients and carers:	
	o It disguised the consumption of food [47];	
	o Patients become emotionally attached to and physically reliant on nasogastric feeding, and were anxious about the tube being removed [47]	
	o Used as a form of punishment and seen as a strategy that doctors used to assert their control [47];	
	o It was easier to avoid nutrition rehabilitation [47];	
	o “*NG feeding becomes enmeshed as an integral and valued sense of patients personal identity or if it becomes entwined with a desire to preserve a public status as an anorexic*” which may contribute to the patient valuing AN more highly than recovery. It is a personal and public signifier of AN [47];	
	o “….my lasting memory of being fed by a tube was that it was very very intrusive” [27];	
	o Two parents believed that the tube was kept in for too long, which made the reintroduction of solid foods more difficult [27].	
**Parenteral nutrition**		
**Benefits**	**Adverse effects**	
• It requires minimal patient cooperation [31].	• It may reinforce a tendency to focus only on physical symptoms rather then the psychiatric implications of AN [31];	
	• Sabotage occurs by pouring solutions into the sink and removing the device [8,31];	
	• It cannot teach patients anything about eating, food choice or portion size, or to perceive their bodies more accurately [31].	
	• Medical complications i.e. infections, arterial injury, cardiac arrhythmias (from placement), changes in vascular endothelium, hyper-osmolarity, and hyperglycaemia [44]; hypophosphataemia and hypokalemia [8];	
	• More medically intensive [31,44,50];	
	• Financial cost [8,44].	

## Discussion

The initial aim of this paper was to undertake a meta-analysis of all papers describing the feeding methods used for the inpatient treatment of AN, to determine which feeding method was the most effective, and to document occurrence of any adverse effects in the samples examined. As the majority of studies (84.6%) were observational cohorts and retrospective chart reviews, and much of the content of these papers were based on descriptions of practice rather than being based on data obtained from a robust research method, it was not possible to complete a meta-analysis.

While there is much to be learnt from clinical expertise it is impossible to determine the superiority of any feeding methods based on this alone. Instead, an attempt was made by the authors to extract data from papers that met the inclusion criteria on sample size, age, admission and discharge weight, weight gain during treatment, length of stay and energy prescribed during treatment. This task was a difficult one, due in most cases to the data being presented in a non-standardised or inconsistent way that made it impossible to compare the results between papers. For example, 17 papers reported admission weight in kilograms, 31 reported admission BMI, seven reported percentage of ideal body weight, and only seven reported height measurements. Some papers reported more than one weight variable, for example, admission weight in kilograms and admission BMI. Discharge weight variables were reported in 31 of 37 samples, however 20 reported discharge weight in kilograms, 21 reported discharge BMI, and four reported percentage of ideal body weight. For the 33 samples that reported a weight change variable over the course of treatment, weight change was reported in kilograms for 24 samples, three reported weight change in grams per day, and seven reported the change in BMI. It could be argued that these variables (i.e. sample size, age, admission and discharge weight/BMI, weight gain during treatment, length of stay and energy prescribed) be mandatory in any research describing inpatients with AN to further knowledge of the effectiveness of treatment.

All feeding methods, apart from six papers that used food only, were mixed feeding methods. That is, the method of feeding was a combination of oral food intake as meals and snacks, and liquid supplements, nasogastric feeding or parenteral nutrition. The majority of papers described the use of a second feeding method, in addition to food, to increase the intake of energy to achieve weight gain. High-energy liquid supplements were used to supplement meals and snacks if the prescribed amount of food was not consumed at the meal [20]. Nasogastric feeding was also used overnight to contribute extra energy [33,34] in addition to what was consumed during the day. Pertushuk, et al. (1983) described the same rationale for the use of parenteral nutrition in achieving additional energy for weight gain, with parenteral nutrition discontinued once the patient was eating adequate amounts [31]. Diamanti, et al. (2008) described a similar process for the use of parenteral nutrition with infusions tapered off as voluntary oral intake increases [8].

The authors’ recommendation is that a randomised prospective study (with sufficient power) be undertaken exploring different feeding methods. It should collect all the necessary data to make an informed decision about the relative effectiveness of each feeding method. We note that good clinical practice may include mixed methods and as such this should be seen as a feeding method and described in detail to allow for appropriate comparisons.

The largest numbers of participants were treated with food only (n = 1189), and notably with less disadvantages reported when compared to nasogastric feeding (n = 560) and parenteral nutrition (n = 173) (Table 3). Most concerns with nasogastric feeding (Table 3) were in regards to medical complications such as reflux, throat pain, nasal irritation, a bleeding nose, the effects of tube tampering, and damage to the psychological engagement with patients. In one study, 55% of patients removed the tube, and all but three removed it five or more times with repeated insertions of tube increasing the risk of complications [27]. Central device infections while being re-fed were one of the main concerns with parenteral nutrition [8].

No deaths were reported in the nasogastric, food only and liquid supplement samples, however four deaths were reported in samples that were treated with parenteral nutrition and food. However, these deaths cannot necessarily be attributed to the method, as Tonoike, et al. (2004) reported that two out of 51 participants died after inpatient treatment when they were followed up 25 months after discharge [36]. An additional death was reported in this sample on day nine of treatment after being transferred from another hospital [36]. Another death was reported from the refeeding syndrome in Weinsier, et al. (1981) [51].

While nasogastric feeding and parenteral nutrition appear to have more adverse effects than food only and liquid supplements, it is not possible to determine whether this is a result of the feeding method or resulting from another factor. For example, it is possible that subjects treated with nasogastric feeding or parenteral nutrition were more unwell, had greater severity of illness, or were less motivated to eat food orally. In most papers, confounding factors were not discussed in consideration of the results presented. For example, in most papers there was no discussion of the role of meal supervision in ensuring that nutrients that are prescribed are actually ingested. Only two papers were identified in this literature search, which described the benefits of meal supervision, and defined the processes involved [18,52]. Notably, Leichner (2005) found that when meal supervision was implemented on an adolescent eating disorder specialist unit, the rates of nasogastric feeding reduced [52].

The best that could be achieved from this review was a description of benefits versus adverse effects of the feeding methods, which are summarised in Table 3. In single studies, some authors have concluded that the described feeding method was a superior method, however, these conclusions are not supported by this current analysis. For example, Rigaud, et al. (2007) concluded that nasogastric feeding is superior to other feeding methods [9]. The study demonstrated slightly better short-term rate of weight gain with non significant differences at 12 month follow up in reference to; percent of patients relapsing, those patients maintaining a BMI > 18.5 kg/m^2^, higher energy intake and total score on the Eating Disorder Inventory. However, there were no differences between the nasogastric feeding and food only groups in regards to psychological recovery, satisfaction with treatment or medical complication frequency. This paper did demonstrate that nasogastric feeding is equally safe as food only feeding owing to the lack of reported medical complications in this study.

The study by Zuercher (2003) showed differences in mean weight gain between 226 receiving food only compared to 84 patients receiving nasogastric feeding (0.82 versus 1.0 kg per week) and delivery of more energy via nasogastric feeding [38]. However, the length of stay for the nasogastric feeding group was longer (61 versus 48 days). Both methods were effective in achieving the National Institute of Clinical Excellence recommendations of 0.5 to 1.0 kg/week in the short term [2]. One of the advantages of nasogastric feeding is that it can be a life saving option [27,53] as it can be implemented if a patient refuses nutrition orally, and it enables clinicians to take control of treatment in life threatening situations. Whether nasogastric feeding should be used as standard treatment or only in life saving or medically compromised patients warrants further examination because of reports from patients that they experienced harm from this method [47].

Diamanti, et al. (2008) compared 104 food only feeding patients and 94 parenteral nutrition patients and found no significant differences with respect to weight gain, recovery and rehospitalisation rate [8]. There were however, more medical complications in the parenteral nutrition sample (Table 3); and an increased cost of parenteral nutrition at 1.7 times more expensive than food only feeding [8]. There have been three subsequent publications disagreeing with this author about the routine use of parenteral nutrition [54-56]. Melchior and Corcos (2009) commented that parenteral nutrition offers less support to the immune and gastrointestinal systems than enteral nutrition, leading to higher rates of infection, impairment of gastric emptying and colonic motility [54].

## Conclusion

Based on the results of this literature review, there is limited evidence on the efficacy of feeding methods used in the refeeding and nutritional rehabilitation of AN, therefore no conclusion can be made about the most effective method of achieving weight gain during inpatient treatment. Considering the importance of re-nutrition in the overall treatment of AN, this is a concern. While there are a number of papers exploring this issue there is no consistency in the way the information is reported to enable comparisons between the different methods. There is an urgent need for research in this area to guide decision-making, as currently little research directs clinicians towards best practice. The literature does provide direction for further research which can be achieved if clinicians working in the area work collaboratively to further knowledge and understanding of refeeding and nutritional rehabilitation in AN. The summary of benefits and adverse effects in Table 3 needs to be observed with caution, and is not necessarily a guide that can be extrapolated to clinical practice.

In order to improve our knowledge of feeding methods in AN, there should be a minimum data set reported on research describing inpatient treatment for AN that includes admission and discharge BMI (or weight and height), weight change during treatment, and length of stay. Additionally, details of the feeding regime, such as nutrient composition and energy delivered would be useful. The authors also recommend that the short term benefits of feeding methods used in the treatment of AN, such as weight gain, and long term benefits, such as recovery rate of participants, be examined using a robust study design.

### Limitations

It is a limitation that only English articles were reviewed. Additionally, it was difficult to extract papers from the literature search using key words, as there was diverse usage and no consistent keywords used, which made identifying useful papers difficult. Some authors have published a number of papers, and it is unclear whether the papers describe the same or different cohorts.

## Abbreviations

AN: Anorexia nervosa; BMI: Body mass index; kg: Kilograms; kg/m2: Kilograms per metre squared; kg/week: Kilograms per week; kCal: Kilocalories; NG: Nasogastric feeding; PN: Parenteral nutrition; SUP: High-energy liquid supplements; RCT: Randomised controlled trial.

## Competing interests

The authors declare that they have no financial or non-financial competing interests.

## Authors’ contributions

SH – Completed literature search; analysed papers and determined which papers met inclusion and exclusion criteria; wrote and reviewed manuscript. RF - Analysed papers and determined which papers met inclusion and exclusion criteria; wrote and reviewed manuscript. SA - Wrote and reviewed manuscript. JR - Wrote and reviewed manuscript. All authors read and approved the final manuscript.
